# Prevalence of chronic kidney disease across levels of glycemia among adults in Pudong New Area, Shanghai, China

**DOI:** 10.1186/1471-2369-14-253

**Published:** 2013-11-16

**Authors:** Yi Zhou, Justin B Echouffo-Tcheugui, Jian-jun Gu, Xiao-nan Ruan, Gen-ming Zhao, Wang-hong Xu, Li-ming Yang, Hong Zhang, Hua Qiu, K M Venkat Narayan, Qiao Sun

**Affiliations:** 1 Pudong New Area Center for Disease Control and Prevention, 3039 Zhang Yang Road, Shanghai 200136, China; 2 Hubert Department of Global Health, Rollins School of Public Health, Emory University, Atlanta, GA, USA; 3 Health Bureau of Shanghai Pudong New Area, 820 Cheng Shan Road, Shanghai 200125, China; 4 Department of Epidemiology, School of Public Health, Fudan University, 138 Yi Xue Yuan Road, Shanghai, China; 5 Key Laboratory of Public Health Safety, Ministry of Education (Fudan University), 138 Yi Xue Yuan Road, Shanghai 200032, China

**Keywords:** Chronic kidney disease, Glycemia, Epidemiology

## Abstract

**Background:**

Few population-based studies have examined the relationship between glycemic status and chronic kidney disease (CKD) in China. We examined the prevalence of CKD across categories of glycemia [diagnosed diabetes, undiagnosed diabetes (fasting plasma glucose [FPG] ≥ 126 mg/dL), prediabetes (FPG 100–126 mg/dL) and normal glycemia (FPG <100 mg/dL)] among Chinese adults and assessed the relative contribution of dysglycemia (prediabetes and/or diabetes) to the burden of CKD.

**Methods:**

5,584 Chinese adults aged 20–79 years were selected from the Pudong New Area of Shanghai through a multistage random sampling. Demographic and lifestyle characteristics, anthropometry and blood pressure were measured. Biochemical assays included FPG, serum creatinine and lipids, urinary creatinine and albumin. Prevalence of albuminuria [urine albumin-to-creatinine ratio (ACR) ≥ 30 mg/g], decreased kidney function and CKD (either decreased kidney function or albuminuria) across levels of glycemia were estimated.

**Results:**

The prevalence of albuminuria, decreased kidney function and CKD each increased with higher glycemic levels (*P* < 0.001). Based on the MDRD Study equation, the unadjusted CKD prevalence was 30.9%, 28.5%, 14.1% and 9.2% in those with diagnosed diabetes, undiagnosed diabetes, prediabetes and normoglycemia, respectively. The corresponding age-, gender- and hypertension-adjusted CKD prevalence were 25.8%, 25.0%, 12.3% and 9.1%, respectively. In a multivariable analysis, the factors associated with CKD were hypertension (Odds ratio [OR] 1.70, 95% confidence interval [CI]: 1.42-2.03), dysglycemia (OR 1.65, 95% CI: 1.39-1.95), female gender (OR 1.48, 95% CI: 1.25-1.75), higher triglycerides (OR 1.14, 95% CI: 1.08-1.20 per mmol/L), higher body mass index (OR 1.08, 95% CI: 1.05-1.10 per kg/m2), and older age (OR 1.02, 95% CI: 1.01 -1.03 per year). The population attributable risks (PARs) associated with diabetes, prediabetes, dysglycemia (diabetes and prediabetes) and hypertension were 18.4%, 19.7%, 30.3% and 44.5% for CKD as defined by the MDRD study equation, and 15.8%, 24.4%, 29.2% and 10.0% with the CKD-EPI equation. Estimates of prevalence and ORs of the relative contribution of various risk factors to CKD obtained with the CKD-EPI equation were similar.

**Conclusions:**

As much as 30% of the CKD burden may be associated with dysglycemia among Chinese adults, independent of age, gender and hypertension status. Prevention and control of diabetes and prediabetes should be a high priority in reducing the CKD burden in China.

## Background

Chronic kidney disease (CKD) is increasingly prevalent worldwide, in developing and developed countries alike [1], and is associated with a substantial burden of mortality, morbidity and health care costs [2]. Diabetes mellitus is a growing pandemic [3] and is the leading cause of CKD in many countries, contributing to increased CKD-related morbidity and mortality [4,5]. In the United States, for example, diabetes accounts for 30 to 40% of CKD [6]. Furthermore, recent evidence suggests an increased risk of CKD even in non-diabetic ranges of glycemia [7,8].

In China, the reported prevalence of CKD ranges from 11.0 to 13.0% depending on geography, and diabetes is a potential major contributor to this burden [9-12]. China has the largest number of people living with diabetes in the world [3], with a national prevalence of diabetes and prediabetes estimated at 9.7% (92.4 million adults) and 15.5% (148.2 million adults), respectively [13]. Despite this heavy burden, unlike in some regions of the world where population-based studies have examined the relationship between glycemic status and CKD [14-16] as well as the relative contribution of dysglycemia (prediabetes or diabetes) to CKD worldwide, very few of such investigations have been conducted in China [17].

We therefore estimated the prevalence of CKD across categories of glycemia (diagnosed diabetes, undiagnosed diabetes, prediabetes, or normal glucose tolerance) and assessed the relative contribution of dysglycemia to the frequency of CKD in a representative sample of Chinese adult adults, in the Pudong New Area of Shanghai in China.

## Methods

### Design and participants

A total of 6,387 adults aged 20 to 79 years and residing in the Pudong New Area of Shanghai- China, were randomly selected through a three-stage sampling process to participate in a cross-sectional study conducted between April and July 2008 [18]. Sampling was conducted follows. Based on the residents’ average social economic status, the 30 streets in the Pudong New Area were classified into three groups (each with 10 streets), and 4 streets were randomly selected from each of these groups, making a total of 12 streets. Then, a total of 34 communities, which hold about 71,000 eligible residents, were randomly selected from the 783 communities in the 12 selected streets. The expected number of participants in each community was calculated as 9.0% of its eligible population; and thereafter participants were randomly selected from households in each of the included community. Pregnant women, as well as physically or mentally disabled persons were excluded. Of the 6,387 eligible individuals, 5,584 (87.4%) responded and were interviewed, 804 (12.6%) declined to be interviewed for miscellaneous reasons. The study protocol was approved by Fudan University Institutional Review Board (IRB00002408, FWA00002399).

### Measurements

After obtaining written consent, a structured in-person interview was conducted by trained personnel to collect information on demographic factors, history of hypertension, diabetes and dyslipidemia, use of tobacco and alcohol, physical activity, and family history of hypertension and diabetes. Blood pressure, body weight, standing height, and waist and hip circumferences were measured according to a standardized protocol. Body mass index (BMI) was calculated as weight in kilograms divided by height in meters squared (kg/m^2^) [19], with overweight defined as an adult BMI ≥24 kg/m^2^ and obesity ≥28 kg/m^2^ by the Working Group on Obesity in China [20]. All participants provided 10 mL of fasting blood and 50 mL of morning void urine for biochemical analyses at the People’s Hospital of Shanghai in Pudong New Area. An automatic Biochemical Analyzer (HITACHI 7170A, Hitachi, Ltd, Tokyo, Japan) was used to measure fasting plasma glucose (FPG), serum triglycerides (TG), total cholesterol (TC) and creatinine; urinary concentrations of creatinine and albumin were measured by enzymology or immunoradiometry methods. The inter-assay coefficient of variation was < 1.5% for FPG, < 1.6% for TG, < 3.0% for TC, < 2.1% for serum creatinine, < 7.0% for urinary album, and < 2.1% for urinary creatinine.

### Definitions

Diagnosed diabetes was defined by the answer “yes” to the question, “Have you ever been told by a doctor or other health professional that you have diabetes or sugar diabetes?” People who answered “no” (n = 3380) or were uncertain about their status (n = 1852) were classified, based on FPG, as having either undiagnosed diabetes (FPG ≥ 126 mg/dL), prediabetes (FPG 100–126 mg/dL) or normoglycemia (FPG < 100 mg/dL) [21].

Self-reported hypertension was defined by the answer “yes” to the question, “Have you ever been told by a doctor or other health professional that you have hypertension or high blood pressure?” Undiagnosed hypertension was defined as measured systolic blood pressure ≥ 140 mmHg and/or diastolic blood pressure ≥ 90 mmHg. Prescription of angiotensin –converting enzyme inhibitors (ACEIs) or angiotensin II receptor blockers (ARBs) for treating hypertension and CKD was also ascertained by questionnaire. Hypertension was defined as either self-reported hypertension (including those under medication) or undiagnosed hypertension.

Renal function was defined on the basis of estimated glomerular filtration rate (eGFR), calculated using either the Modification of Diet in Renal Disease (MDRD) equation (modified for Chinese adults) or the Chronic Kidney Disease- Epidemiology Collaboration (CKD-EPI) equation. The MDRD equation is eGFR (mL/min/1.73 m^2^) = 175 × Calibrated – Scr (mg/dL)^−1.234^ × age (year) ^−0.179^ [female × 0.79] [22]. The CKD-EPI equation is eGFR (mL/min/1.73 m^2^) = 141 × min(Scr/ê, 1)^á^ × max (Scr/ê, 1)^-1.209^ × 0.993^Age^ × 1.018 [if female] × 1.159 [if black] [23]; ê is 0.7 for females and 0.9 for males, á is −0.329 for females and −0.411 for males, min indicates the minimum of Scr/ê or 1, and max indicates the maximum of Scr/ê or 1. Decreased kidney function was defined as an eGFR of less than 60 mL/min/1.73 m^2^. Microalbuminuria and macroalbuminuria were defined as a urine albumin–to -creatinine ratio (ACR, mg/g) between 30 to 299 mg/g and ≥ 300 mg/g, respectively, according to theAmerican Diabetes Association (ADA) guidelines [6]. CKD was defined as either decreased kidney function (low eGFR) or kidney damage (albuminuria) or both [24] Albuminuria was measured cross-sectionally, hence we used a modified definition of stages of CKD, which were as follows: stage 1- kidney damage with normal or increased GFR (eGFR ≥ 90 mL/min/1.73 m^2^); stage 2- kidney damage with mild decreased GFR (eGFR 60 to 89 mL/min/1.73 m^2^); stages 3- moderately decreased GFR (eGFR 30 to 59 mL/min/1.73 m^2^); stages 4 or 5- severely decreased eGFR or kidney failure (eGFR < 30 mL/min/1.73 m^2^) [25].

### Statistical analysis

Demographic and clinical characteristics were compared across all four categories of glycemia using the Chi-square tests for categorical variables, and Kruskal-Wallis tests for continuous variables that were not normally distributed. Crude prevalence of CKD, decreased kidney function and albuminuria by glycemic status were calculated. The prevalence of CKD, adjusted for age, gender and hypertension status by glycemic status, was estimated using multiple logistic regression. The odds ratios (ORs) and 95% confidence intervals (CIs) of the independent association between dysglycemia (prediabetes or diabetes) and CKD, adjusting for age, gender, hypertension status, BMI and TG, were estimated using logistic regression models. We used the adjusted ORs to estimate the population attributable risk percent (PAR%), the proportion of the condition/disease in the population due to the presence of risk factors or the proportion of the condition/disease in the population that would be eliminated if the risk factors for CKD were removed. The PAR% was calculated for diabetes, prediabetes or both conditions relative to normoglycemia (used as referent group), using the Levin’s formula [26]: PAR% = p (r-1)/(p(r-1) +1), where p is the proportion with diabetes, prediabetes or both conditions and r is the odds ratio. With the Levin’s formula, PAR% was also calculated for hypertension relative to normotension (used as referent group). All statistical analyses were done using SPSS version 18 (2010 SPSS Inc; IBM, Chicago, IL).

## Results

### Characteristics of participants by glycemic status

The crude prevalence of diagnosed diabetes, undiagnosed diabetes, prediabetes, and normal glucose tolerance were 6.3%, 4.9%, 24.6% and 64.2%, respectively. Table 1 shows the characteristics of participants (n = 5,584- 2,477 males and 3,107 females) by glycemic status. Compared with those with prediabetes or normal glucose tolerance, people with diagnosed or undiagnosed diabetes were more likely to be older, had a lower education level, be overweight or obese, and be more-frequent drinkers. Men accounted for more than half of the population with undiagnosed diabetes but for less than half of the other categories of glycemia (*P* < 0.001, Table 1). Higher levels of height, weight, TG or TC and lower levels of eGFR (using the MDRD or CKD-EPI equation) were observed among individuals with undiagnosed diabetes compared to those with other glycemic status. ACR was higher among those with diagnosed or undiagnosed diabetes than among those with prediabetes or normoglycemia. The reported percentage of people taking ACEI/ARBs was overall low, but relatively higher in those with diagnosed diabetes.

**Table 1:** Characteristics of participants by glycemic status

**Characteristic**	**Glycemic status**				***P***
	**Diagnosed diabetes**	**Undiagnosed diabetes**	**Prediabetes**	**Normoglycemia**	
*n(%)*	346 (6.7)	274 (5.3)	1360 (26.2)	3557 (68.5)	—
Age (years)	59.0 (53.0,68.0)	57.0 (49.0,65.3)	55.0 (48.0,62.0)	49.0 (37.0,57.0)	<0.001
Gender (%)					<0.001
Male	46.5	53.3	47.1	42.5	
District (%)					0.360
Rural	62.1	55.5	57.9	57.8	
Height (cm)	162.0 (155.0,167.1)	162.5 (156.0,170.0)	162.5 (157.0,169.0)	162.0 (157.0,169.0)	0.019
Weight (kg)	65.0 (57.5,73.0)	68.0 (60.0,74.0)	65.1 (58.0,72.5)	61.0 (55.0,69.0)	<0.001
FPG (mg/dL)	141.3 (118.4,174.1)	148.3 (134.0,199.8)	106.2 (102.5,111.6)	91.1 (86.8,94.9)	<0.001
TG (mg/dL)	186.0 (132.9,292.3)	212.6 (141.7,318.9)	177.1 (115.1,265.7)	141.7 (97.4,212.6)	<0.001
TC (mg/dL)	189.5 (162.4,216.6)	197.2 (174.0,224.3)	189.5 (166.3,216.6)	177.9 (154.7,201.1)	<0.001
eGFR (ml/min/1.73 m^2^)					
MDRD Study–estimated	107.7 (88.4,125.2)	110.6 (90.4,128.6)	101.1 (87.9,118.6)	108.0 (93.1,125.1)	<0.001
CKD-EPI–Estimated	94.9 (81.9,103.6)	96.4 (83.4,106.6)	95.0 (83.6, 103.9)	100.7 (89.5,111.6)	<0.001
ACR (mg/g)	13.2 (4.8,37.0)	11.7 (4.8,36.8)	6.9 (2.6,16.5)	5.8 (2.2,12.1)	<0.001
BMI (kg/m, %)					<0.001
<24	39.0	32.1	41.8	59.7	
Overweight, 24-	44.5	47.1	43.2	31.8	
Obesity, ≥ 28	16.5	20.8	15.0	8.5	
Hypertension (%)	60.3	54.7	36.6	20.9	<0.001
ACEIs/ARBs (%)	1.4	0.7	0.4	0.2	0.002
Education (%)					<0.001
Less than high school	72.8	71.9	65.9	56.1	
High school or more	27.2	28.1	34.1	43.9	
Household income per person year *in Yuan* (%)					0.398
<12,000	54.9	58.0	56.5	58.6	
12,000–	42.5	40.9	40.8	38.7	
36,000–	2.0	0.0	2.0	1.8	
≥72,000	0.6	1.1	0.7	0.9	
Smoking (%)					0.112
Sometimes/not at all	74.0	70.8	77.1	74.9	
Every day	26.0	29.2	22.9	25.1	
Alcohol drinking (%)					<0.001
Nondrinkers	23.2	22.1	27.7	35.2	
1-2 drinkers per week	18.9	17.6	15.4	20.0	
3-4 drinkers per week	7.4	7.4	12.6	11.0	
5-6 drinkers per week	15.8	8.8	7.5	6.6	
everyday drinkers	34.7	44.1	36.8	27.2	

Data expressed as median (25th, 75th percentile) for continuous variables and percentage for categorical variables. *P* value for Kruskal-Wallis test (continuous variables) or *χ*^*2*^ test (categorical variables). Diagnosed diabetes, self-reported of diabetes diagnosis; undiagnosed diabetes, FPG **≥** 126 mg/dL and no self-report of diabetes; prediabetes, FPG **≥** 100 and **<** 126 mg/dL and no self-report of diabetes; no diabetes, FPG **<** 100 mg/dL and no self-report of diabetes.

*Abbreviations*: *TG* Triglyceride, *TC* Total cholesterol, *eGFR* Estimated glomerular filtration rate, *ACR* Albumin-creatinine ratio, *CKD-EPI* Chronic kidney disease epidemiology collaboration, *MDRD* Modification of diet in renal disease.

### Prevalence of albuminuria, decreased kidney function and CKD by glycemic status

Table 2 presents data on the prevalence of kidney damage. The crude prevalence of albuminuria, decreased kidney function and CKD differed significantly across the glycemic groups. Albuminuria was present in 28.9% in people with diagnosed diabetes, 28.1% of those with undiagnosed diabetes, 12.9% of those with prediabetes, and 8.7% of those with normoglycemia (*P* < 0.001). Regardless of the method of GFR estimation, those with diabetes or prediabetes had a higher prevalence of decreased kidney function than those with normoglycemia (3.5%, 1.8%, 2.6% and 0.9% with the MDRD Study equation, and 6.1%, 2.9%, 4.1% and 1.6% with the CKD-EPI equation, for diagnosed, undiagnosed diabetes, prediabetes and normoglycemia, respectively, *P* < 0.001). The unadjusted CKD prevalence using the MDRD Study equation was 30.9%, 28.5%, 14.1% and 9.2% in those with diagnosed diabetes, undiagnosed diabetes, prediabetes and normoglycemia, respectively (*P* < 0.001). The prevalence of CKD (at any stage) was significantly higher in individuals with diabetes or prediabetes than in those with normoglycemia (*P* < 0.05). Meanwhile, the prevalence of CKD stages 1 and 2 was higher than the prevalence of stage 3 through 5 in individuals across glycemic categories (*P* < 0.05). Among those with CKD, as defined by the MDRD equation, 15.2% had diagnosed diabetes, 11.1% had undiagnosed diabetes and 27.3% had prediabetes. Similar proportions were observed with the use of CKD-EPI equation - 15.4%, 10.8% and 27.6% for diagnosed, undiagnosed diabetes and prediabetes, respectively. With the CKD-EPI equation, the unadjusted CKD prevalence was somewhat higher (33.6%, 29.2%, 15.2% and 9.8% for diagnosed, undiagnosed diabetes, prediabetes and normoglycemia, respectively) but showed a similar pattern across the glycemic categories with slightly more individuals in stage 2 or stage 3 of CKD (Table 2).

**Table 2:** Kidney function by glycemic status, with estimation of GFR by the MDRD Study equation and the CKD-EPI equation

**Characteristic**	**Glycemic status**								***P***
	**Diagnosed diabetes**		**Undiagnosed diabetes**		**Prediabetes**		**Normoglyciemia**		
	**n**	**Prevalence (95% CI)**	**n**	**Prevalence (95% CI)**	**n**	**Prevalence (95% CI)**	**n**	**Prevalence (95% CI)**	
Albuminuria (%)	100	28.9 (24.1-33.7)	77	28.1 (22.8-33.5)	176	12.9 (11.2-14.7)	308	8.7 (7.8-9.6)	<0.001
Microalbuminuria (%)	81	23.4 (18.9-27.9)	71	26.0 (20.8-31.2)	162	12 (10.3-13.8)	296	8.4 (7.5-9.3)	<0.001
Macroalbuminuria (%)	19	5.5 (3.1-7.9)	6	2.2 (0.5-4.0)	14	1.0 (0.5-1.6)	12	0.3 (0.2-0.5)	<0.001
MDRD Study–estimated									
Decreased kidney function (%)	12	3.5 (1.5-5.4)	5	1.8 (0.2,3.4)	35	2.6 (1.7,3.4)	31	0.9 (0.6,1.2)	<0.001
CKD (%)	107	30.9 (26.0-35.8)	78	28.5 (23.1-33.8)	192	14.1 (12.3-16.0)	326	9.2 (8.2-10.1)	<0.001
CKD Stage 1	73	6.1 (3.5-8.6)	63	3.7 (1.4-5.9)	113	3.2 (2.3-4.2)	230	1.7 (1.3-2.2)	0.002
CKD Stage 2	21	6.1 (3.5-8.6)	10	3.7 (1.4-5.9)	44	3.2 (2.3-4.2)	62	1.7 (1.3-2.2)	
CKD Stage 3	8	2.3 (0.7-3.9)	4	1.5 (0.0-2.9)	33	2.4 (1.6-3.3)	24	0.7 (0.4-0.9)	
CKD Stage 4/5	2	0.6 (0.2,1.4)	0	0.0 (0.0-0.0)	0	0.0 (0.0-0.0)	2	0.1 (0.0-0.1)	
CKD-EPI–Estimated									
Decreased kidney function (%)	21	6.1 (3.5-8.6)	8	2.9 (0.9-4.9)	56	4.1 (3.1-5.2)	58	1.6 (1.2-2.1)	<0.001
CKD (%)	114	33.6 (28.5-38.5)	80	29.2 (23.8-34.6)	204	15.2 (13.4-17.2)	342	9.8 (8.8-10.8)	<0.001
CKD Stage 1	63	18.2 (14.1-22.2)	52	19.0 (14.3-23.7)	96	7.1 (5.7-8.4)	208	5.9 (5.1-6.6)	<0.001
CKD Stage 2	37	10.7 (7.4-14.0)	25	9.1 (5.7-12.6)	80	5.9 (4.6-7.1)	100	2.8 (2.3-3.4)	
CKD Stage 3	13	3.8 (1.7-5.8)	3	1.1 (0.2-2.3)	28	2.1 (1.3-2.8)	34	1.0 (0.6-1.3)	
CKD Stage 4/5	1	0.3 (0.2-0.9)	0	0.0 (0.0-0.0)	0	0.0 (0.0-0.0)	0	0.0 (0.0-0.0)	

Data presented as n, percentage (CI, confidence interval) for categorical variables. *P* value for *χ*^*2*^ test (categorical variables).

Albuminuria was defined as a urinary albumin-to-creatinine ratio (ACR) >30 mg/g creatinine; Microalbuminuria and macroalbuminuria were defined as a ACR between 30 and 299 mg/g and 300 mg/g or greater, respectively. CKD was defined as estimated glomerular filtration rate(eGFR) < 60 mL/min/1.73 m^2^ or albuminuria.

*Abbreviations*: *CKD* Chronic kidney disease, *CKD-EPI* Chronic kidney disease epidemiology collaboration, *MDRD* Modification of diet in renal disease.

Adjustment for age, gender, and hypertension status resulted in a slightly lower CKD prevalence [25.8%, 25.0%, 12.3% and 9.1% for diagnosed diabetes, undiagnosed diabetes, prediabetes and normoglycemia, respectively] but similar patterns across the diabetes categories, using either the MDRD Study or the CKD-EPI equation (Table 3). In pair-wise comparisons, the adjusted CKD prevalence using the MDRD Study equation was higher in those with diagnosed diabetes than in those undiagnosed (25.8% vs. 25.0%), and higher in those with prediabetes than in those with normoglycemia (12.3% vs. 9.1%) (*P* < 0.008). When using the CKD-EPI equation, although the adjusted CKD prevalence appeared lower figures obtained with the MDRD equation (overlapping confidence intervals), individuals with diagnosed or undiagnosed diabetes (Table 3) still had a higher burden of CKD compared to those with prediabetes or normoglycemia (24.5%, 23.3%, 12.1% and 9.8%, for diagnosed, undiagnosed diabetes, prediabetes and normoglycemia, respectively).

**Table 3:** Adjusted prevalence of CKD by glycemic status and selected characteristics, with estimation of GFR by the MDRD Study equation and the CKD-EPI equation (%)

**Characteristic**	**Glycemic status**				***P***
	**Diagnosed diabetes**	**Undiagnosed diabetes**	**Prediabetes**	**Normoglycemia**	
MDRD Study–estimated					
Overall	25.8 (21.0-31.2)	25.0 (19.9-30.9)	12.3 (10.3-14.6)	9.1 (4.2-13.9)	<0.001
Age (years)					
20-59	23.8 (17.7-31.2)	27.9 (21.0-36.0)	10.4 (8.3-13.0)	7.7 (4.8-9.6)	<0.001
≥60	28.5 (21.3-37.0)	22.6 (15.4-31.9)	15.8 (12.0-20.6)	13.1 (6.9-11.6)	<0.001
Gender					
Male	23.4 (16.9-31.4)	21.1 (14.8-29.1)	8.6 (6.3-11.5)	6.7 (3.1-9.5)	<0.001
Female	23.8 (17.9-30.9)	25.5 (18.5-34.2)	14.5 (11.7-17.9)	11.6 (7.0-14.2)	<0.001
BMI (kg/m^2^)					
<24	23.3 (16.5-31.8)	22.5 (14.7-32.9)	9.5 (7.0-12.6)	7.1(2.7-14.2)	<0.001
Overweight, 24-	25.4 (18.5-33.9)	24.4 (17.2-33.5)	12.5 (9.5-16.2)	11.0(3.6-22.2)	<0.001
Obesity, ≥28	27.2 (16.4-41.6)	30.5 (18.8-45.4)	19.4 (13.2-27.5)	18.5(3.5-45.4)	0.002
Hypertension					
Yes	35.1 (27.6-43.4)	35.4 (26.6-45.5)	17.3 (13.5-21.8)	16.7 (6.4-36.8)	<0.001
No	19.9 (14.0-27.6)	20.1 (14.0-28.0)	10.5 (8.2-13.3)	6.9 (3.4-13.3)	<0.001
CKD-EPI–Estimated					
Overall	24.5 (20.0-29.7)	23.3 (18.4-29.0)	12.1 (10.2-14.3)	9.8 (5.0-13.8)	<0.001
Age (years)					
20-59	23.8 (17.7-31.2)	28.0 (21.1-36.1)	10.4 (8.3-13.0)	7.8 (5.0-9.4)	<0.001
≥60	33.2 (25.6-41.9)	24.1 (16.7-33.4)	18.7 (14.6-23.7)	15.7 (8.7-14.0)	<0.001
Gender					
Male	23.5 (17.1-31.4)	20.4 (14.3-28.2)	9.1 (6.8-12.1)	7.3 (3.4-10.4)	<0.001
Female	24.4 (18.5-31.5)	24.5 (17.7-32.8)	14.3 (11.5-17.5)	11.7 (7.1-14.4)	<0.001
BMI (kg/m^2^)					
<24	23.7 (17.0-32.1)	20.6 (13.3-30.4)	9.4 (7.1-12.4)	7.5 (3.0-15.0)	<0.001
Overweight, 24-	25.7 (18.8-34.1)	24.9 (17.7-33.9)	13.0 (10.0-16.8)	11.5 (3.8-23.2)	<0.001
Obesity, ≥28	30.4 (18.8-45.1)	30.4 (18.8-45.4)	21.2 (14.7-29.7)	19.5 (3.7-36.8)	0.001
Hypertension					
Yes	37.5 (29.8-45.9)	36.9 (28.0-46.8)	18.9 (15.0-23.6)	18.0 (7.1-30.9)	<0.001
No	20.8 (14.8-28.4)	19.2 (13.3-26.8)	10.9 (8.6-13.7)	7.5 (3.8-14.2)	<0.001

Data presented as percentage (95%CI, confidence interval) for categorical variables. *P* value for *χ*^*2*^ test (categorical variables). Prevalence estimates were adjusted for age, gender and hypertension status using multivariable logistic regression, excluding variables being examined (e.g., age-stratified prevalence adjusted for gender and hypertension status only). CKD was defined as estimated glomerular filtration rate (eGFR) < 60 mL/min/1.73 m^2^ or albuminuria.

*Abbreviations*: *CKD* Chronic kidney disease, *BMI*, Body mass index, *MDRD* Modification of diet in renal disease, *CKD-EPI* Chronic kidney disease epidemiology collaboration.

### Relative contribution of dysglycemia to the burden of CKD

**Figure 1:**
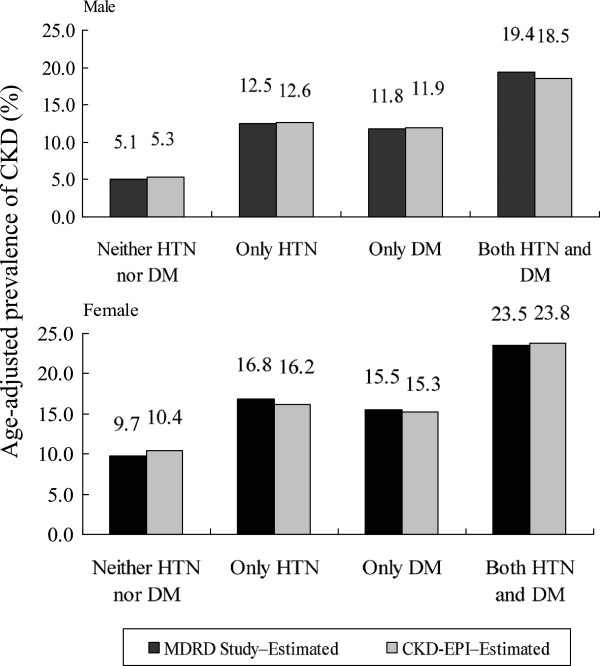
**Comparison of age-adjusted prevalence of CKD with estimation of the GFR by the MDRD Study equation and the CKD-EPI equation, stratified by hypertension or hyperglycemic status.** CKD prevalence expressed as mean ± SD. All *P* values less than 0.05 for ANOVA tests across all four groups. HTN refers to the self-reported patients, those with the use of antihypertensive drugs, or detected with undiagnosed hypertension; DM refers to self-reported type 2 diabetes or elevated FPG (FPG ≥ 100 mg/dL). Abbreviations: *MDRD* Modification of diet in renal disease, *CKD-EPI* Chronic kidney disease epidemiology collaboration, *FPG* Fasting plasma glucose, *HTN* Hypertension.

Figure 1 shows data on the relative contribution of dysglycemia (diabetes or prediabetes) and hypertension to the burden of CKD, through a comparison of the age-adjusted prevalence of CKD among individuals with only hypertension, only dysglycemia, both, and neither of these conditions. Regardless of the equation used to derive eGFR, the highest age-adjusted CKD prevalence was consistently observed in those with hypertension and dsyglycemia (19.4% in men and 23.5% in women using the MDRD Study equation; 18.5% in men and 23.8% in women using the CKD-EPI equation). Those with only hypertension or only dysglycemia had a lower prevalence of CKD, and those with neither of these conditions had the lowest prevalence (*P* < 0.05). Women had a higher prevalence of CKD than men, in any of the hypertension and/or dysglycemia groups (*P* < 0.05).

As shown in Table 4, MDRD-defined CKD was independently associated with hypertension (OR 1.70, 95% CI: 1.42-2.03), dysglycemia (OR 1.65, 95% CI: 1.39-1.95), female gender (OR 1.48, 95% CI: 1.25-1.75), higher TG (OR 1.14, 95% CI: 1.08-1.20 per mmol/L), higher BMI (OR 1.08, 95% CI: 1.05-1.10 per kg/m^2^), and older age (OR 1.02, 95% CI: 1.01 -1.03 per year). Albuminuria was also associated with increased age, female sex, higher BMI, higher TG, hypertension and dysglycemia, while decreased GFR was associated with increased age, male sex, and hypertension. The ORs obtained using the CKD-EPI equation were consistent with the results obtained with the MDRD Study equation (Table 4). The PAR% for CKD (defined using the MDRD Study equation) was 18.4%, 19.7%, 30.3% and 44.5% for diabetes, prediabetes, dysglycemia and hypertension, respectively. The corresponding figures of PAR% based on the CKD-EPI equation were 15.8%, 24.4%, 29.2% and 10.0%, respectively.

**Table 4:** Risk factors for kidney damage and CKD - multivariable logistic regression analysis

	**Albuminuria**	**Decreased GFR**		**CKD**	
		**MDRD Study–estimated**	**CKD-EPI–estimated**	**MDRD Study–estimated**	**CKD-EPI–estimated**
Age	1.01 (1.01,1.02)	1.10 (1.07,1.12)	1.14 (1.12,1.17)	1.02 (1.01,1.03)	1.03 (1.02,1.04)
Gender (female vs male)	1.62 (1.36,1.92)	0.58 (0.37,0.91)	0.89 (0.63,1.27)	1.48 (1.25,1.75)	1.50 (1.27,1.76)
BMI (kg/m^2^)	1.07 (1.05,1.10)	1.07 (1.00,1.14)	1.02 (0.97,1.08)	1.08 (1.05,1.10)	1.07 (1.04,1.10)
TG (mg/dL)	1.14 (1.08,1.21)	1.01 (0.80,1.28)	1.06 (0.89,1.25)	1.14 (1.08,1.20)	1.15 (1.09,1.22)
Hypertension	1.70 (1.41,2.04)	2.20 (1.34,3.60)	1.72 (1.18,2.50)	1.70 (1.42,2.03)	1.64 (1.38,1.95)
Diabetes or prediabetes	1.69 (1.42,2.02)	1.48 (0.93,2.36)	1.30 (0.91,1.87)	1.65(1.39,1.95)	1.60 (1.36,1.89)

Data are multivariable-adjusted odds ratios (95% CI). Hypertension was defined as systolic blood pressure **≥**140 mm Hg or diastolic blood pressure **≥**90 mm Hg, the use of antihypertensive drugs, or by any self-reported history of hypertension. Diabetes or prediabetes referred to self-reported type 2 diabetes or elevated FPG (FPG **≥** 100 mg/dL).

*Abbreviations*: *GFR* Glomerular filtration rate, *MDRD* Modification of diet in renal disease, *CKD-EPI* Chronic kidney disease epidemiology collaboration, *TG* Triglyceride, *FPG* Fasting plasma glucose.

## Discussion

Our study shows an important contribution of dysglycemia to the occurrence of CKD, irrespective of the approach used to defining CKD. We found that CKD prevalence, as estimated by low eGFR and/or the presence of albuminuria, was higher among individuals with diabetes (diagnosed and undiagnosed) or prediabetes compared with normoglycemia, regardless of CKD stage and GFR estimating equation. Indeed, around a third of people with diabetes and 15.0% of those with prediabetes had CKD. Furthermore, microalbuminuria, a marker of endothelial injury and a harbinger for progression to CKD [27], was present in 23.4% of our participants with diabetes. Dysglycemia independently explained 30.3% of the PAR of CKD, while hypertension explained 44.5%.

The prevalence of albuminuria and CKD was high among people with diagnosed (28.9%) and undiagnosed (28.1%) diabetes and was similar to that found in recent population-based studies from other parts of Asia [17,28,29]. The prevalence of CKD among people with diabetes was, however, lower than that found in many community-based or primary care studies [15,30,31]. but higher that that reported in a recent community-based study in Taiwan [32]. The overall prevalence of CKD observed in our study was similar to that reported in other North American, European and Asian countries (10.5–13.1%) [9,30,33,34]. where the increase in CKD has been attributed to a number of risk factors including diabetes, hypertension, and age of 60 or greater [35,36].

The high contribution of dysglycemia to the occurrence of CKD found in our study reflects the rapid rise in the burden of diabetes mellitus in China over the recent decades. Of the participants with CKD, 15.2% had diagnosed diabetes, 11.1% had undiagnosed diabetes and 27.3% had prediabetes. Hitherto, the role of various risk factors to the occurrence of CKD in developing countries has not been clearly defined. In China, although evidence on CKD based on renal biopsies had previously indicated that primary glomerulonephritis (GN) was the most common form of renal diseases [37], the latter condition has declined considerably in the past two decades, while the incidence of diabetic nephropathy has increased significantly [12]. While it is clear from studies mainly including Caucasians that diabetes is a strong risk factor for developing CKD and end-stage renal disease [8,38], very few studies have examined the contribution of prediabetes, a more prevalent condition than diabetes, to the occurrence of CKD. In our study, the prevalence of CKD in people with prediabetes was lower than the age-, gender-, ethnicity-adjusted 17.1% observed in the 1999–2006 National Health and Nutrition Examination Survey (NHANES) in the United States [15]. However, our prevalence of CKD among people with dysglycemia was higher than observed in a screening study for diabetes complications among Chinese from two urban communities in Shanghai (crude prevalence of CKD was 23.6% in subjects with diabetes and impaired glucose regulation) [17]. These differences may be related to demographic characteristics and the methods of diabetes ascertainment, as nearly half of diabetes cases in our study were diagnosed through biochemical testing, while most cases in other studies were self-reported [39]. Interestingly, people with prediabetes had a relatively high prevalence of decreased kidney function, similar to those with diagnosed diabetes, indicating that the decrease in GFR may start early in the natural history of diabetes, and may be due to hyperfiltration state in the early stages of hyperglycemia. Consequently, albuminuria and eGFR may have complementary roles in screening different age groups, and the use of these two variables together could be proved more efficient at identifying people at a high risk of progression to kidney failure [40].

The method of GFR estimation may influence the figures of CKD prevalence; hence we used two commonly accepted equations. The prevalence of CKD obtained with the MDRD Study equation was relatively low compared to that obtained in other populations [15,39]. However, the MDRD Study equation is the most widely used equation worldwide [41,42]. and has been modified and validated for the Chinese population [9,43]. Though the CKD-EPI equation is said to be a more accurate tool than the MDRD equation, it has not been commonly used in the Chinese population [23,44], and the question on the validity of the GFR estimation using the CKD-EPI equation in this population remains to be answered. Although CKD prevalence obtained with the CKD-EPI equation were higher CKD than that based on MDRD Study equation, which is not consistent with the findings in some previous report [23], the patterns of CKD prevalence across glycemic categories and the direction and magnitude relative contribution of dysglycemia were similar with both GFR estimation equations, confirming the robust relationship between hyperglycemia and CKD.

Our study has some limitations. The cross-sectional design and the lack of oral glucose –tolerance test made it challenging to account for the full spectrum of dysglycemia when evaluating the association between glycemic status and CKD. It is possible that the contribution of non-diabetic hyperglycemia is in fact higher than what we observed, as many people with impaired glucose tolerance may have been misclassified as having normal glucose regulation. There may have been some misclassification of early-stage CKD as a result of the limitations in GFR estimation and single spot urine measurements. Some transient albuminuria was also possible, especially among women who may have urinary tract infections or are menstruating. The duration of diabetes was unknown in our study; longer exposure to hyperglycemia could increase the risk for CKD. A prospective follow-up of our participants is ongoing to further assess the incidence of CKD in people at different levels of glycemia.

Our study has a number of strengths. First, the study includes a large and representative sample of the general population, with a high response rate, and rigorously standardized methods for data collection with stringent quality control. Second, we examined the independent contribution of a large spectrum of hyperglycemia to CKD, including non-diabetic range hyperglycemia. Our findings are very informative, and have important clinical and public health relevance for early detection, prevention and control of CKD among Chinese adults. Indeed, the high CKD prevalence in individuals with dysglycemia regardless of their hypertension status suggests that individuals with prediabetes may be an appropriate target group for CKD screening [45]. Detection of prediabetes may also make CKD screening more effective, and campaigns to promote awareness of both kidney damage and decline in kidney function at this early stage of dysglycemia — targeted at both physicians and the community — may be beneficial [46].

## Conclusions

In summary, dysglycemia appears as a strong, independent contributor to the burden CKD in the Chinese population of Shanghai, a findings that will have to be confirmed in longitudinal studies. In view of the continuous increase of hyperglycemic conditions in China, and in the context of a high prevalence of undetected prediabetes/diabetes and CKD, screening for both prediabetes and diabetes among people with CKD, and screening for CKD among individuals with prediabetes or diabetes, should be considered, as is currently recommended by the American Diabetes Association [47].

## Abbreviations

CKD: Chronic kidney disease; ESRD: End-stage renal disease; FPG: Fasting plasma glucose; TG: Serum levels of triglycerides; TC: Total cholesterol; ACR: Albumin-to-creatinine ratio; ADA: American diabetes association; eGFR: estimated glomerular filtration rate; CKD-EPI: Chronic kidney disease epidemiology collaboration; MDRD: Modification of diet in renal disease; BMI: Body mass index.

## Competing interests

The authors confirm that there are no known conflicts of interest associated with this publication and there has been no significant financial support for this work that could have influenced its outcome.

## Authors’ contributions

JJG, QS, GMZ conceived and designed the study. YZ, XNR, HZ, HQ, LMY performed the investigation. YZ and WHX analyzed the data. YZ wrote the paper. JBE contributed to statistical analysis and critical revision of the paper. KMN contributed to conceptualization of study question, planning of analysis and to revision of the manuscript. All authors read and approved the final manuscript.

## Pre-publication history

The pre-publication history for this paper can be accessed here:

http://www.biomedcentral.com/1471-2369/14/253/prepub
